# An improved YOLOv8n with multi-scale feature fusion for real time and high precision railway track defect detection

**DOI:** 10.3389/frai.2025.1711309

**Published:** 2026-01-09

**Authors:** Zhihong Zhang, Liling Zhang, Xin Lu, Tingting Ma, Feng Huang, Sheng Zhong

**Affiliations:** 1 Guangzhou Institute of Metrology and Testing Technology, Guangzhou, China; 2 School of Artificial Intelligence and Automation, Huazhong University of Science and Technology, Wuhan, China

**Keywords:** lightweight model, multi-scale feature fusion, rail defect detection, real-time detection, YOLOv8

## Abstract

**Introduction:**

Railway transportation is increasingly critical for modern urban and intercity mobility. However, the expanding scale and intensifying operational intensity of rail networks have elevated track defect detection to a key concern. Traditional inspection methods (manual, ultrasonic, eddy current, magnetic flux leakage testing) are limited by insufficient accuracy, low efficiency, or poor adaptability to complex environmental conditions.

**Methods:**

An enhanced defect detection framework based on an improved YOLOv8 algorithm was proposed, tailored for small targets and complex backgrounds. Three core improvements were integrated: 1) AVCStem module with variable convolution kernels to dynamically adapt to defects of different shapes and scales; 2) ADSPPF module using multi-scale pooling and multi-branch attention mechanisms to preserve fine-grained features across scales; 3) MSF module for enhanced multi-scale feature fusion via partial convolution and hierarchical feature alignment.

**Results and Discussion:**

Experiments on a real-world track defect dataset showed the proposed model achieved 90.2% detection precision, 90.2% mAP@0.5, and 73.2% mAP@0.5:0.95. Meanwhile, the model size was reduced to 5.2MB with 2.45M parameters. Comparative and ablation studies confirmed the complementary advantages of each module and the model’s superior performance over existing lightweight detectors. The proposed model provides a robust, accurate, and efficient solution for real-time railway defect detection. It exhibits strong potential for deployment in edge AI devices and mobile inspection robots, addressing the limitations of traditional inspection methods.

## 1 Introduction

As an efficient and convenient mode of transportation in modern society, rail transportation has undergone rapid global development in recent years. Various types of rail systems, including urban subways, light railways, high-speed railways, and heavy-haul railways, have continually expanded their network mileage and enhanced transportation capacity to meet the growing demands for population mobility and economic exchange (Xiong et al., 2023; Guerrieri et al., 2018). However, with the expanding scale of rail transit networks and the increasing intensity of operations, ensuring safe and stable functioning has become a critical concern. As the foundational infrastructure that directly supports train movement, the track structure is subjected to prolonged exposure to dynamic and static loads, environmental degradation, and material fatigue, which frequently results in various structural defects (Kou, 2022; Fu et al., 2023). Without timely and accurate detection and repair, such defects can escalate into catastrophic failures, including increased track irregularities, train derailments, and overturns, thereby endangering passenger safety and causing substantial economic losses and adverse social impacts (Zhong and Chen, 2024). Currently, traditional railway inspection methods include manual inspection (Rahman et al., 2024), ultrasonic testing (Wang et al., 2023), eddy current testing (Alvarenga et al., 2021), and magnetic flux leakage (MFL) testing (Tang et al., 2021). Although manual inspection is straightforward, it suffers from subjectivity and inspector fatigue, leading to inconsistent results and high labor costs (Yaodong et al., 2024; Kumar and Harsha, 2025). Ultrasonic detection, while cost-effective and technologically mature, operates slowly and requires coupling agents, resulting in limited effectiveness on coated or uneven surfaces (Yuan et al., 2024). Eddy current testing is highly sensitive to surface and near-surface defects and does not require a coupling agent, yet it performs poorly in detecting deep-seated defects (Wang et al., 2022). MFL detection features simple equipment and fast scanning but exhibits low accuracy and sensitivity to defect geometry.

In recent years, advancements in deep learning have brought substantial breakthroughs in computer vision algorithms. Object detection algorithms can be broadly categorized into two types: region-based fully convolutional networks (R-FCN)(Tang et al., 2020), and spatial pyramid pooling networks (SPPNet) (Han et al., 2020), and end-to-end detection frameworks such as single shot multibox detector (SSD) (Bai et al., 2021), You Only Look Once (YOLO) (Wang et al., 2024), and detection transformer (DETR) (Gibert et al., 2016). Region proposal-based approaches extract potential target regions within the image and process each region individually for classification and bounding box refinement (Chen et al., 2023). In contrast, end-to-end methods eliminate region proposals and directly predict object classes and positions from the entire image, significantly improving detection speed (Wu et al., 2020; Yin et al., 2022).

In this study, a dataset of track images collected from real-world railway environments was constructed, targeting surface damage, missing fasteners, missing bolts, and other anomalies for model training and evaluation. However, due to the small size of the defects, complex background interference, and limited feature representation, the YOLOv8 algorithm demonstrates a high false detection rate in this context. To address these issues, this study proposes an improved YOLOv8-based framework incorporating several architectural enhancements:

- (a) To improve the backbone network’s capacity to extract features from defects of varying sizes, a variable convolution kernel (AKConv) is embedded within the VoV-GSCSP module, forming a new AKConv-VoV-GSCSP Stem (AVCStem) to replace the original C2f module. This modification dynamically adjusts the receptive field during feature aggregation, effectively handling rail cracks of different orientations and accurately locating missing components under conditions of strong light reflection.
- (b) To mitigate fine feature loss caused by increased network depth and repeated downsampling, the SPPF module is extensively redesigned based on the SENetV2 architecture. A progressive multi-scale pooling strategy is introduced, resulting in the ADSPPF module, which retains features ranging from micro-cracks to large-scale fastener loss.
- (c) To further enhance the transmission of small-scale features, this study proposes a refined multi-scale feature fusion neck network. By integrating a weighted feature fusion strategy with a bidirectional feature pyramid structure, a new multi-scale fusion module (MSF) is developed to improve the model’s ability to capture fine-grained defect details.

## 2 YOLOv8

**Figure 1:**
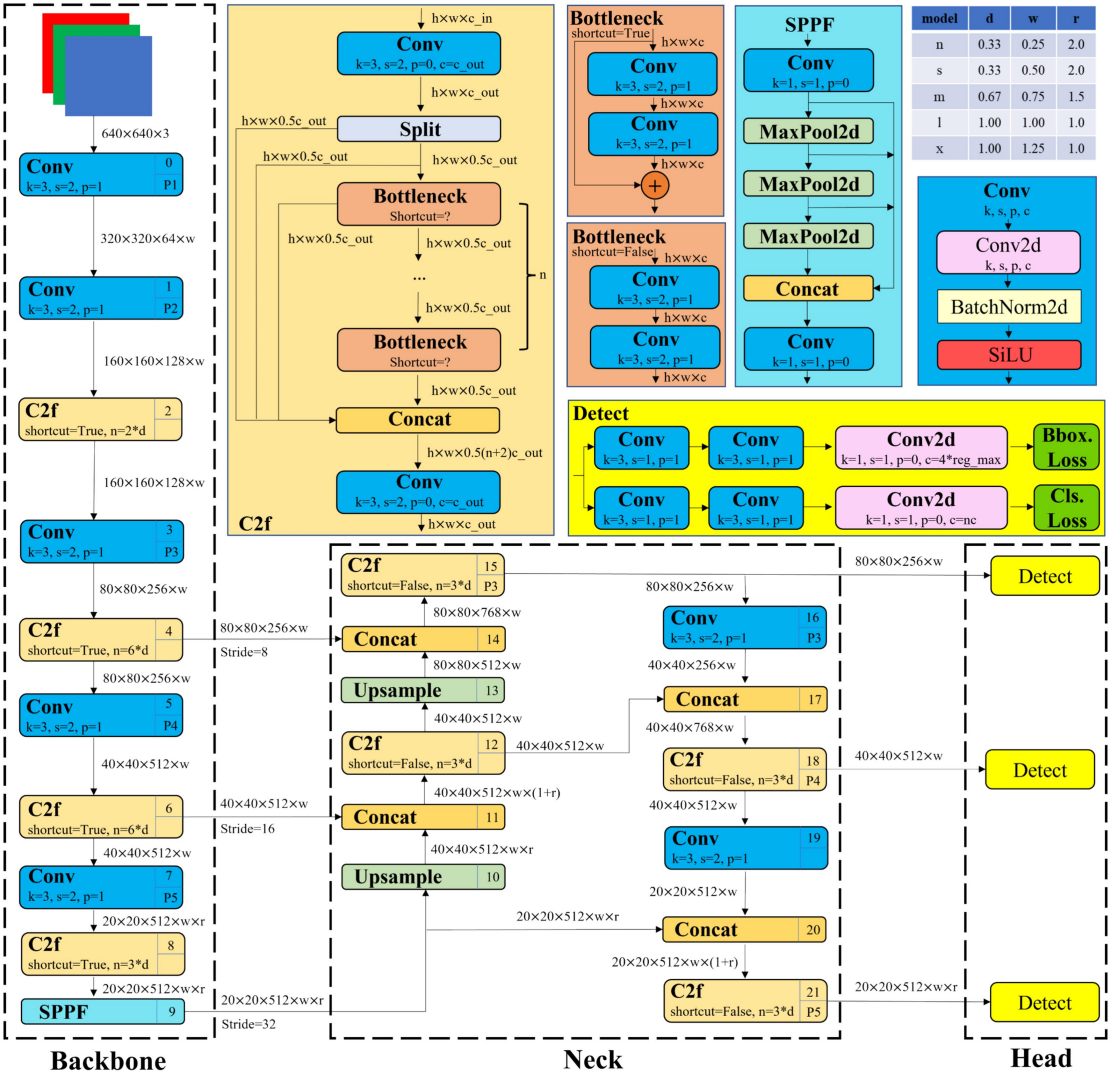
The structure of YOLOv8 (Terven et al., 2023).

As a well-recognized variant of the YOLO series proposed by Ultralytics, YOLOv8 achieves substantial improvements in accuracy and inference speed compared with earlier-generation models, making it a commonly used baseline in related studies. In terms of network architecture, it retains the classic three-stage structure of “Backbone-Neck-Head,” but has implemented multiple optimizations in module design and connection methods, further enhancing detection accuracy and speed. Its structure is as shown in Figure 1.

YOLOv8 accepts 3-channel color images with a resolution of 640 × 640. The Backbone network consists of Conv, C2f, and SPPF modules. The Conv module is used for image feature extraction and dimension adjustment; the C2f module captures gradient flow information and enhances feature extraction capability through Bottleneck units. The SPPF module fuses contextual information of different scales through pooling layers of multiple scales, enhances the receptive field, and maintains computational efficiency at the same time. The Neck adopts the Path Aggregation Network-Feature Pyramid Network (PAN-FPN) structure to realize feature fusion, and finally outputs 3 feature maps of different scales to the Head part (Haroon et al., 2024). The Head uses an Anchor-Free design, whose core function is to directly predict the target’s location, confidence, and category based on the fused feature maps. These designs enable YOLOv8 to perform excellently in both real-time detection scenarios and high-precision demand scenarios, making it one of the mainstream models in the current object detection field (Aydin et al., 2021).

YOLOv8n, as the smallest model in the YOLOv8 series, boasts advantages such as fast detection speed and low resource consumption. However, if YOLOv8n is directly applied to the task of rail surface defect detection, the model will face problems such as occlusion, reflection, and poor detection performance for small targets. To address these issues, targeted adjustments to the model are required to enhance its ability to detect targets at different scales, thereby improving the overall performance of rail surface defect detection.

## 3 Improved algorithm design

### 3.1 Optimizing backbone networks

**Figure 2:**
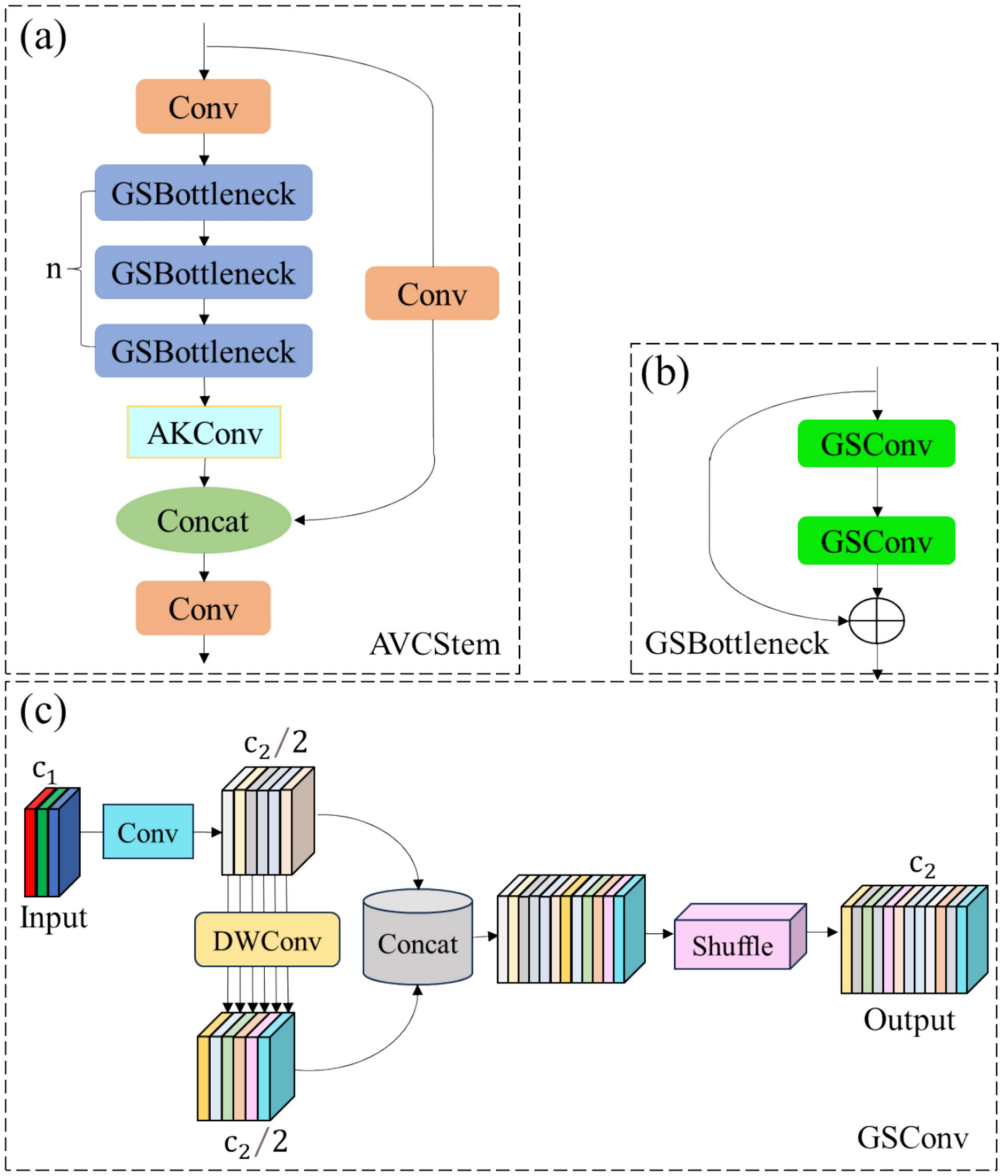
Schematic diagram of the AVCStem structure: **(a)** Overall architecture of the AVCStem module; **(b)** Structure of the GSBottleneck layer; **(c)** Structure of the GSConv layer. In the YOLOv8n backbone network, the original C2f module is replaced by the AVCStem module, which integrates the AKConv variable convolution and the Ghost Bottleneck structure to enhance the robust feature extraction capability for multi-directional cracks and defects in highly reflective areas.

In the field of target detection algorithms, the design of the backbone network plays a key role in the performance of the model. As an efficient target detection model, the C2f module in the backbone network of YOLOv8n plays a certain role in the feature extraction process. However, in order to further improve the feature extraction ability of the model, reduce the number of parameters and enhance the adaptability to targets of different scales, this paper proposes to replace the C2f module in the YOLOv8n backbone network with the AVCStem module. The AVCStem module, as shown in Figure 2a, is a feature extraction module for the backbone network of the target detection model. It combines multiple convolution operations and bottleneck structures to achieve effective extraction and fusion of multi-scale features (Yang et al., 2022; Acikgoz and Korkmaz, 2023). The design of this module aims to make full use of the information in the feature map, while reducing the model parameters and improving the model’s detection ability for targets of different sizes and shapes. The specific structure and workflow of the AVCStem module are as follows: The input feature map X first passes through two standard convolutional layers, which are used to perform preliminary feature transformation and extraction on the input feature map. Then the output feature map of a convolutional layer enters a sequence consisting of multiple grouped ghost bottleneck sequence layers (GSBottleneck). The GSBottleneck layer is a lightweight bottleneck structure, as shown in Figure 2b. It combines group shuffle convolution (GSConv) and residual connection, which can reduce the number of parameters while maintaining good feature extraction capabilities. In the GSBottleneck layer, a 1 × 1 GSConv is first used to reduce the dimension of the input feature map, and then a 3 × 3 GSConv is used for feature extraction (Li et al., 2022). Finally, the input feature map and the output feature map are added through the residual connection to enhance the feature expression ability. Among them, the GSConv structure is shown in Figure 2c, and its efficiency advantage stems from a “channel compression—depthwise enhancement—shuffle fusion” pipeline tailored for track defect feature extraction: (1) The input feature map (channels $c_{1}$) is compressed to $c_{1}$/2 via standard 1 × 1 convolution (retaining cross-channel information while reducing computation base); (2) The compressed feature map is processed by 3 × 3 depthwise convolution (enhancing local defect details like microcrack edges with minimal overhead); (3) The two feature maps from steps (1) and (2) are concatenated and channel-shuffled (eliminating information isolation without extra computation). To quantify this advantage, we compare GSConv with two mainstream lightweight convolutions—standard grouped convolution (SGC, G = 2) and depthwise separable convolution (DSConv)—using unified parameters: input feature map H × W × C_in_, output channels C_out_, and kernel size 3 × 3(consistent with track defect detection settings).

This design allows GSConv to significantly reduce computational overhead while maintaining cross-channel feature interaction—critical for distinguishing tiny track defects (e.g., 1–2 pixel microcracks) from background noise—making it suitable for lightweight models deployed on edge inspection devices.

**Figure 3:**
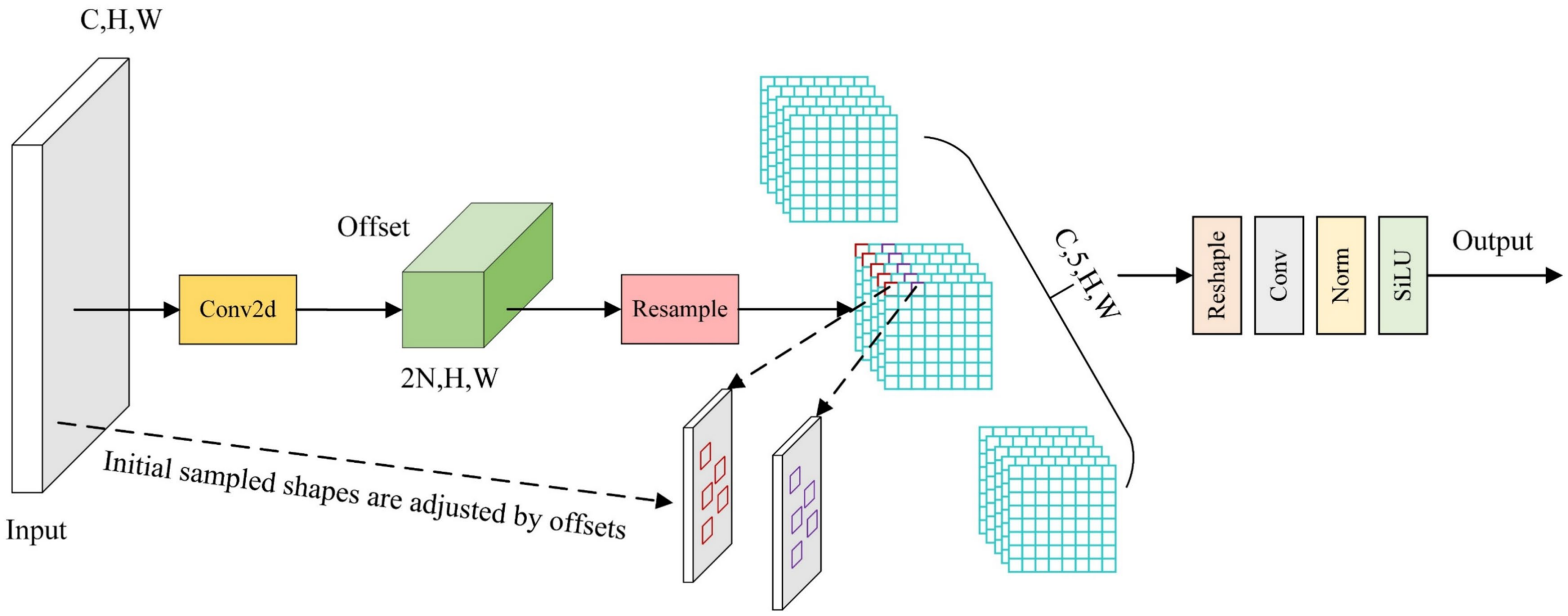
The AKConv structure diagram.

To address the problem that fixed convolution kernels cannot adapt to track defects of different shapes and scales (e.g., irregular cracks, scattered missing bolts, and directional surface damage), this paper proposes the AVCStem module, which integrates GSBottleneck and AKConv (Adaptive Convolution). As shown in Figure 3, AKConv realizes “dynamic adjustment” of sampling positions by learning pixel-level offset vectors, thereby enhancing the extraction of multi-scale defect features.

Specifically, AKConv first constructs a hybrid sampling grid Pn​ consisting of “regular base points” and “irregular adaptive points”: the regular base points (e.g., 9 points for 3 × 3 kernel equivalence) ensure basic feature extraction capability, while the adaptive points (4 per base point, determined by track defect scale statistics) supplement flexibility for irregular defects. To generate dynamic offsets, a lightweight prediction branch is embedded in AKConv: after the input feature map is processed by 1 × 1 convolution (channel reduction) and BatchNorm2d, a 3 × 3 depthwise convolution outputs an offset tensor $\Delta P\in R^{H\times W\times 2K}$ (where H × W is the feature map size, K is the total number of sampling points, and “2” corresponds to x/y-axis offsets). The tanh activation function restricts ΔP to [−1, 1] pixel units to avoid excessive sampling deviations. The final adaptive sampling coordinates are calculated as $P_{\text{final}}=P_{n}+P_{0}+\mathrm{\Delta P}$ ($P_{0}$ is the center of the convolution window), and the convolution operation is defined as Formula 1:


$$\text{Conv}(P_{0})=\sum_{n}\omega \times F(P_{n}+P_{0}+\mathrm{\Delta P})$$ (1)

where *ω* denotes trainable convolution weights, and F(·) is the feature value at the adjusted position (obtained via bilinear interpolation). The offset ΔP is learned end-to-end through the model’s overall detection loss (classification loss + CIoU regression loss + confidence loss), which implicitly guides sampling points to align with defect regions.

Finally, the feature map output by AKConv is concatenated with the feature map output by a convolution layer in the channel dimension, and then a standard convolution is used to perform a convolution operation on the concatenated feature map to adjust the number of channels of the feature map. In summary, the AVCStem module achieves effective extraction and fusion of multi-scale features through a series of convolution operations, grouped convolution bottleneck structure and adaptive convolution. Applying it to the YOLOv8n backbone network to replace the C2f module can make full use of the information in the feature map, reduce model parameters, and improve the model’s feature extraction ability and detection performance for targets of different scales.

### 3.2 Fast aggregation of dense spatial pyramid modules

**Figure 4:**
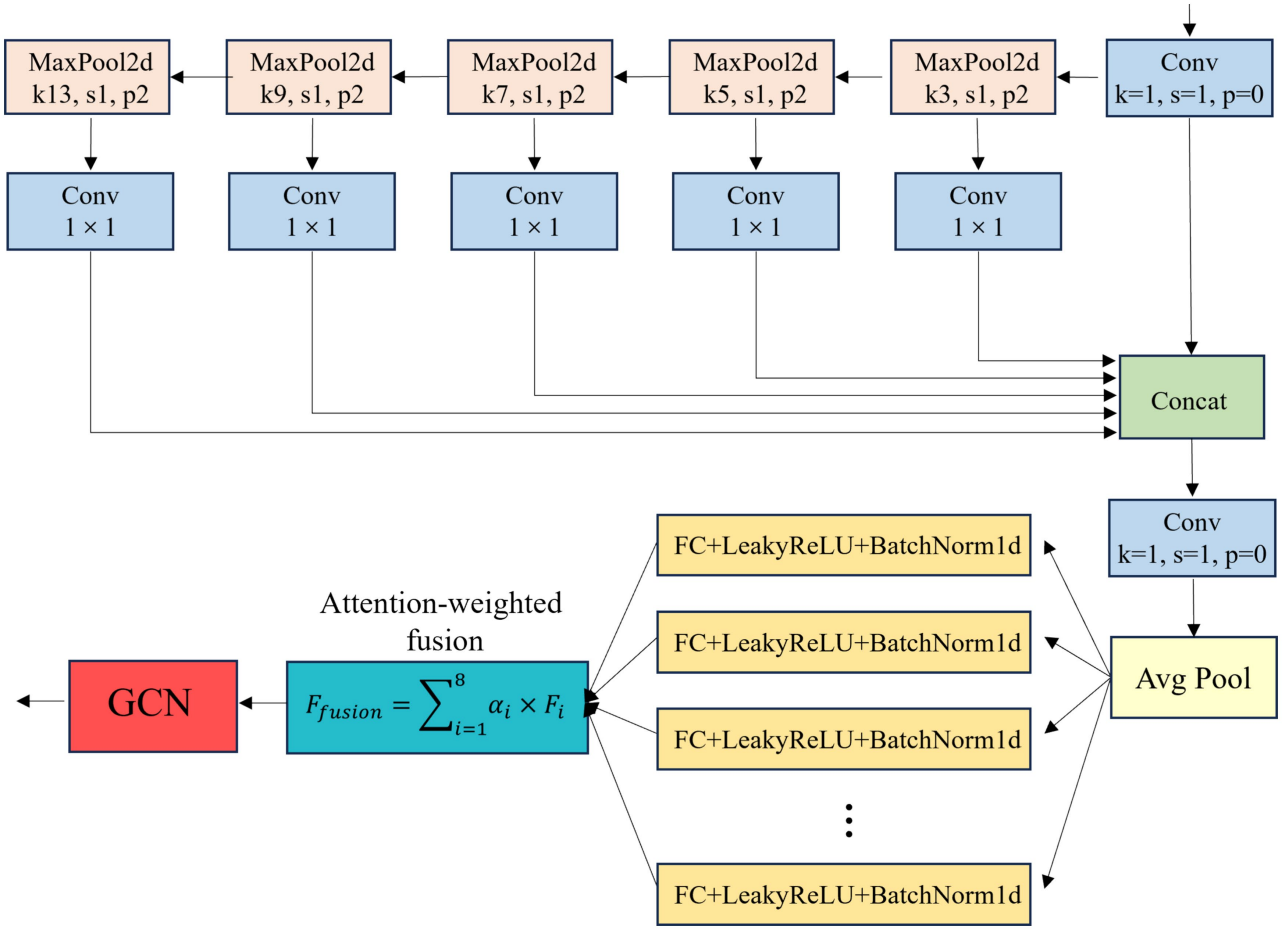
ADSPPF structure. The red dashed boxes denote the progressive pooling branches (3 × 3 → 13 × 13), where pooling operations with incrementally increasing kernel sizes process the same preprocessed feature map (40 × 40 × 256) to capture multi-scale defects; the blue arrows indicate the flow direction of the feature maps after dimension reduction via 1 × 1 convolution (downsampled to 1 × 1 × 128), which are subsequently concatenated and refined by the attention mechanism (green box) to eliminate redundancy.

In the task of railway track defect detection, although the SPPF module of YOLOv8 enables multi-scale feature fusion, its application in real-world railway scenarios still encounters significant challenges. As network depth increases and downsampling operations are repeatedly applied, fine features of small defects (e.g., microcracks on the rail surface and missing bolts) are easily lost, particularly when detecting tiny cracks like rail head damage (Wang et al., 2022; Phaphuangwittayakul et al., 2024). Simultaneously, the similarity between the complex metal texture background and surface damage on the track, combined with illumination interference caused by mirror reflections on the rail, complicates accurate feature extraction (Min et al., 2023). To address this, this study redesigns SPPF based on SENetV2 (Narayanan, 2023) to create the ADSPPF module (Figure 4), which integrates multi-scale progressive pooling, multi-branch attention, and illumination normalization.

#### 3.2.1 Detailed design of ADSPPF

The ADSPPF module processes input feature maps (e.g., 40 × 40 × 512 from P4 layer) in three stages:

(1) Multi-scale progressive pooling. The “progressive” design of ADSPPF refers to applying incrementally sized pooling kernels to the same input feature map within a single module (rather than assigning different kernels to different network layers), ensuring unified capture of multi-scale defect features without cross-layer information loss. Specifically: First, the input feature map (e.g., 40 × 40 × 512 from P4 layer) undergoes a pre-pooling 1 × 1 Conv (Table 1) to compress channels from 512 to 256—this reduces computational redundancy while retaining core defect information, providing a consistent foundation for multi-scale pooling. Next, the preprocessed 40 × 40 × 256 feature map is fed into 5 parallel max-pooling branches with incrementally increasing kernel sizes: 3 × 3 → 5 × 5 → 7 × 7 → 9 × 9 → 13 × 13. Each kernel size is optimized for railway track defect scales (statistically analyzed from the dataset in Section 4.2): small kernels (3 × 3, 5 × 5) preserve fine details of microcracks (1–3 pixels) and surface scratches; medium kernels (7 × 7, 9 × 9) capture moderate defects (e.g., missing buckles); large kernels (13 × 13) integrate contextual information of large-scale defects (e.g., missing fastener clusters). All pooling operations use a stride of 1 and zero-padding to ensure output size remains 1 × 1 (global pooling for each scale), avoiding local feature loss. Each pooling branch is followed by a 1 × 1 Conv layer to reduce channels from 256 to 128—this step filters redundant feature channels (e.g., background metal textures shared across scales) and aligns feature dimensions for subsequent fusion, outputting 5 feature maps of 1 × 1 × 128 each.
(2) Multi-branch attention and feature reorganization. First, the 5 pooled feature maps are fed into 8 parallel fully connected (FC) branches, with branch dimensions following a “base-4” rule: 4, 8, 12, 16, 20, 24, 28, 32 neurons per branch (total dimension = 32). Each branch specializes in learning a specific feature type: e.g., Branch 1 (4 neurons) learns brightness variations, Branch 2 (8 neurons) learns texture features of surface cracks (responsive to small pooling kernels like 3 × 3), and Branch 8 (32 neurons) learns geometric features of missing bolts (responsive to large kernels like 13 × 13); Second, each FC branch uses LeakyReLU (*α* = 0.1) activation to retain negative gradient information (critical for low-contrast defects in shadows) and BatchNorm1d to stabilize training; Last, a learnable attention vector α∈R^8^ (normalized via softmax) weights the output of each branch (Formula 2):

$$F_{\text{fusion}}=\sum_{i=1}^{8}\alpha_{i}\times F_{i}$$ (2)

**Table 1:** ADSPPF module parameter and tensor size details.

Component	Input tensor size	Output tensor size	Parameters (k)	Activation	Normalization
Conv (pre-pooling)	40 × 40 × 512	40 × 40 × 256	131.1	SiLU	BatchNorm2d
Progressive Pooling (3 × 3–13 × 13)	40 × 40 × 256	1 × 1 × 128 (×5)	0	–	–
Multi-branch FC (8 branches)	1 × 1 × 640 (5 × 128)	1 × 1 × 32	20.6	LeakyReLU (α = 0.1)	BatchNorm1d
Attention weight Layer	1 × 1 × 32	1 × 1 × 32	0.032	Softmax	–
Conv (post-fusion)	1 × 1 × 32	40 × 40 × 512	16.4	SiLU	BatchNorm2d
Total	–	–	73.9	–	–

The progressive pooling component (3 × 3 → 13 × 13) introduces no additional parameters (0 k) but requires 0.05G GFLOPs for 5 parallel branches—this computational overhead is offset by the pre-pooling Conv (reducing channels from 512 to 256, saving 0.08G GFLOPs) and post-fusion attention (suppressing redundant features), resulting in a net GFLOPs increase of only 0.15G vs. SPPF (Table 4).

This attention mechanism emphasizes defect-relevant features (e.g., crack edges from small kernels, fastener shapes from large kernels) and suppresses redundant background information—for example, when detecting microcracks, α assigns higher weights to texture-focused branches; when detecting missing fasteners, geometric-focused branches are prioritized.

(3) Illumination invariance realization. GCN and multi-branch attention form a “preprocessing to refinement” synergy to eliminate illumination interference (e.g., rail glare, tunnel shadows) while preserving defect details. Before progressive pooling, the input feature map (40 × 40 × 512 from P4 layer) undergoes GCN (Formula 3):

$$F_{\mathrm{norm}}(x,y)=\frac{F(x,y)-\mu }{\sigma +\in }$$ (3)

where $\mu =\frac{1}{H\times W}\sum_{x,y}F(x,y)$ (global mean), $\sigma =\sqrt{\frac{1}{H\times W}\sum_{x,y}{\left(F(x,y)-\mu \right)}^{2}}$ (global standard deviation), $\epsilon =10^{-6}$. This normalizes the feature map to mean = 0 and standard deviation = 1, suppressing glare-induced brightness saturation and shadow-induced contrast loss—critical for ensuring consistent input to the attention branches.

GCN provides “illumination-invariant” input for the 8 FC branches: it reduces irrelevant illumination outliers (e.g., glare spots normalized from 255 to ~1.2) and enhances defect features (e.g., shadowed microcracks normalized from 50 to ~ − 0.8), allowing the branches to focus on learning defect features rather than compensating for lighting. The attention branches further refine this input by: (1) assigning low $\alpha_{i}$ to residual illumination-related features (via Branches 1–2); (2) assigning high $\alpha_{i}$ to texture/geometric defect features (via Branches 3–8). This synergy is reinforced during training: if residual illumination still interferes with detection, ${ℒ}_{\text{total}}$ increases, and the attention branches are updated to suppress illumination-related $F_{i}$ via gradient backpropagation.

#### 3.2.2 Parameter and tensor size annotation

Table 1 summarizes the ADSPPF module’s components, tensor sizes, and parameters. The total parameter count of ADSPPF is 73.9 k, which is 9.2% higher than SPPF (67.7 k), but provides significant gains in feature retention and illumination robustness. These improvements enable the model to maintain stable detection performance in complex railway environments.

### 3.3 Improved multi-scale feature fusion module

**Figure 5:**
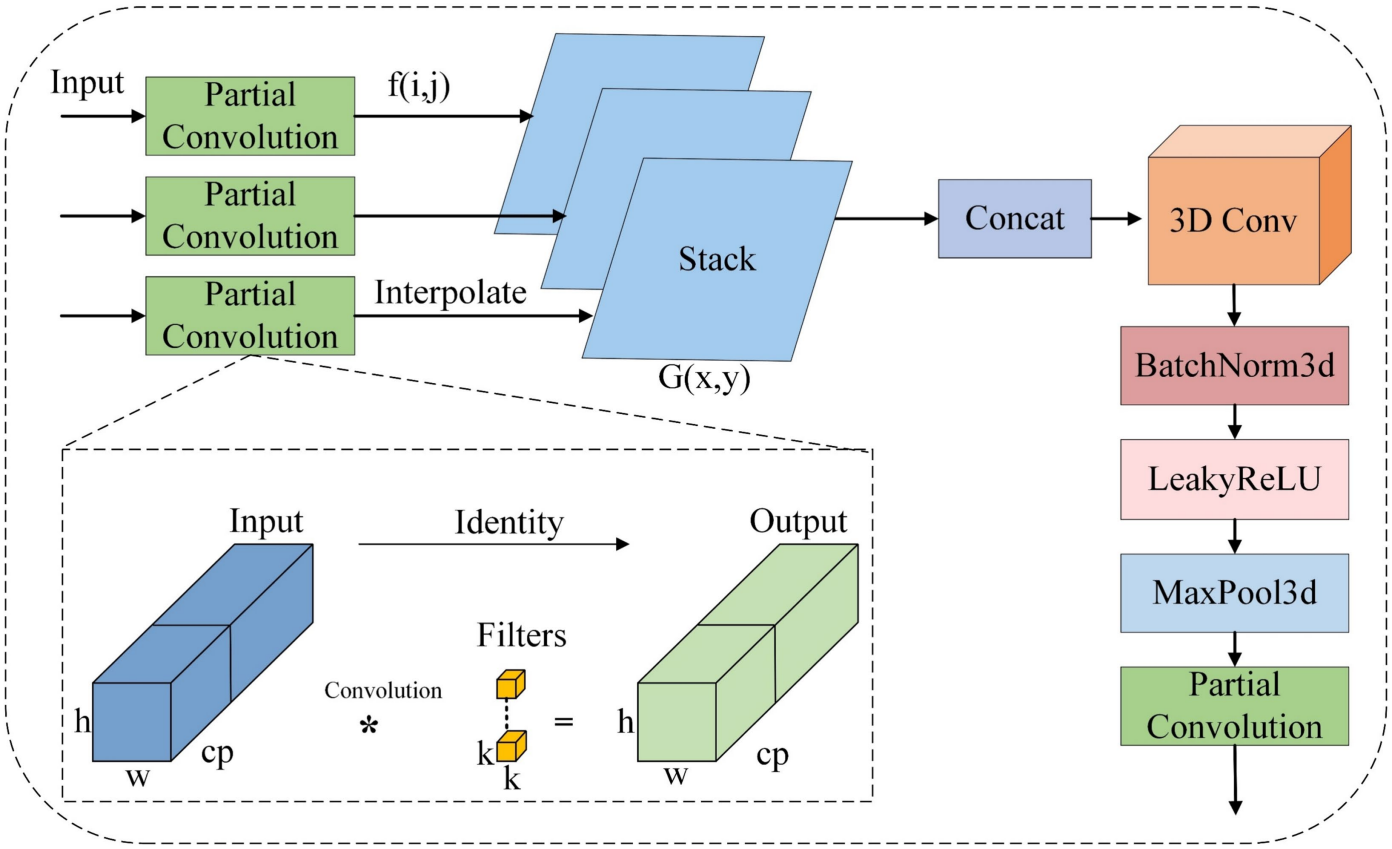
MSF structure. The “*” symbol in the figure represents the convolution operation, through which the input feature map is convolved with the filter to generate a new feature map.

In railway track defect detection, the traditional feature pyramid network (FPN) exhibits limited feature transfer capability for tiny defects—such as microcracks on the rail surface and missing bolts—resulting in insufficient accuracy in small target detection (He et al., 2024). Inspired by the YOLO framework based on attention scale sequence fusion (ASF-YOLO) (Kang et al., 2024), this study introduces a hierarchical feature enhancement mechanism in the neck network of YOLOv8. Simultaneously, to further optimize computational efficiency and reduce redundant feature extraction, partial convolution (PConv) replaces traditional convolution operations at both the front and back ends of the feature enhancement mechanism, and a multi-scale fusion module (MSF) is designed. As shown in Figure 5, this structure adopts a strategy of jointly optimizing spatial and scale features to effectively improve detection accuracy in complex track scenarios while reducing computational complexity.

The MSF module processes feature maps from the backbone (P2: 160 × 160 × 128, P3: 80 × 80 × 256, P4: 40 × 40 × 512) as follows:

(a) Partial convolution (PConv) preprocessing: PConv replaces traditional Conv in FPN to reduce redundant feature extraction, with its core mechanism being “mask-guided partial pixel update”—only valid pixels (defect-related regions) are updated via convolution, while invalid regions (background with low feature relevance) are masked to retain original values. This design avoids mixing defect features with redundant background (e.g., rail ballast, surface reflections) and reduces GFLOPs by 18% compared to standard Conv. The specific implementation for an input feature map $F_{\text{in}}\in R^{H\times W\times C}$ (e.g., P2 layer: 160 × 160 × 128) is as follows.

Step 1: Preliminary feature transformation: A 3 × 3 convolution kernel *ω* is applied to $F_{\text{in}}$ to generate an intermediate feature map $F_{\mathrm{mid}}:F_{\mathrm{mid}}(x,y)=\sum_{u=-1}^{1}\sum_{v=-1}^{1}\omega (u,v)\times F_{\text{in}}(x+u,y+v)$. This step is consistent with traditional convolution and provides a basis for subsequent valid region judgment.

Step 2: Invalid region identification via feature response: To avoid manual threshold tuning, invalid regions are adaptively identified based on the feature relevance of $F_{\text{in}}$.

First, $F_{\text{in}}$ is normalized to [0,1] via min-max normalization, eliminating scale differences between feature values (Formula 4):


$$F_{\mathrm{norm}}(x,y)=\frac{F_{\text{in}}(x,y)-\min(F_{\text{in}})}{\max(F_{\text{in}})-\min(F_{\text{in}})}$$ (4)

The “feature response intensity” S(x,y) (average value across all channels) is calculated to reflect pixel relevance to defects (Formula 5):


$$S(x,y)=\frac{1}{C}\sum_{c=1}^{C}F_{\mathrm{norm}}(x,,y,,c)$$ (5)

Higher S(x,y) indicates stronger correlation with defects (e.g., crack edges), while lower values indicate background. Last, An adaptive threshold T (optimized via cross-validation on the railway dataset) is used to generate a binary mask.

Step 3: Mask-guided partial update: The final output feature map $F_{\text{out}}$ is obtained by fusing the intermediate feature and original feature under mask guidance (Formula 6):


$$F_{\text{out}}(x,y)=M(x,y)\times F_{\mathrm{mid}}(x,y)+(1-M(x,y))\times F_{\text{in}}(x,y)$$ (6)

Valid regions (*M* = 1) retain refined defect features, while invalid regions (*M* = 0) retain original values to avoid redundant updates. For each input feature map (e.g., P2), PConv (*k* = 3, *s* = 1) outputs a refined feature map with the same size, ensuring compatibility with subsequent Gaussian scale smoothing.

(b) Gaussian scale smoothing: Each refined feature map is convolved with a series of Gaussian filters (*σ* = 0.5, 1.0, 1.5, 2.0) to generate multi-scale smoothed features, capturing defect details at different resolutions (Formulas 7, 8):

$$F_{\sigma }(i,j)=\sum_{u}\sum_{v}f(i-u,i-v)\times G_{\sigma }(u,v)$$ (7)

$$G_{\sigma }(x,y)=\frac{1}{2\pi \sigma^{2}}e^{-(x^{2}+y^{2})/2\sigma^{2}}$$ (8)

Where f is the input feature map, and $G_{\sigma }$ is the Gaussian filter with standard deviation σ.

(c) Hierarchical alignment and 3D convolution: Smoothed features from P2, P3, P4 are upsampled to the resolution of P4 (40 × 40) via nearest-neighbor interpolation, then stacked along the channel dimension (output: 40 × 40 × (128 + 256 + 512) × 4, where 4 is the number of Gaussian scales). A 3D Conv layer (k = 3, s = 1) extracts cross-scale and cross-level features, enhancing the representation of small defects.
(d) PConv post-processing: A final PConv layer adjusts the channel count to match the neck’s output requirement (e.g., 40 × 40 × 512), ensuring compatibility with the YOLO Head.

### 3.4 Network structure

**Figure 6:**
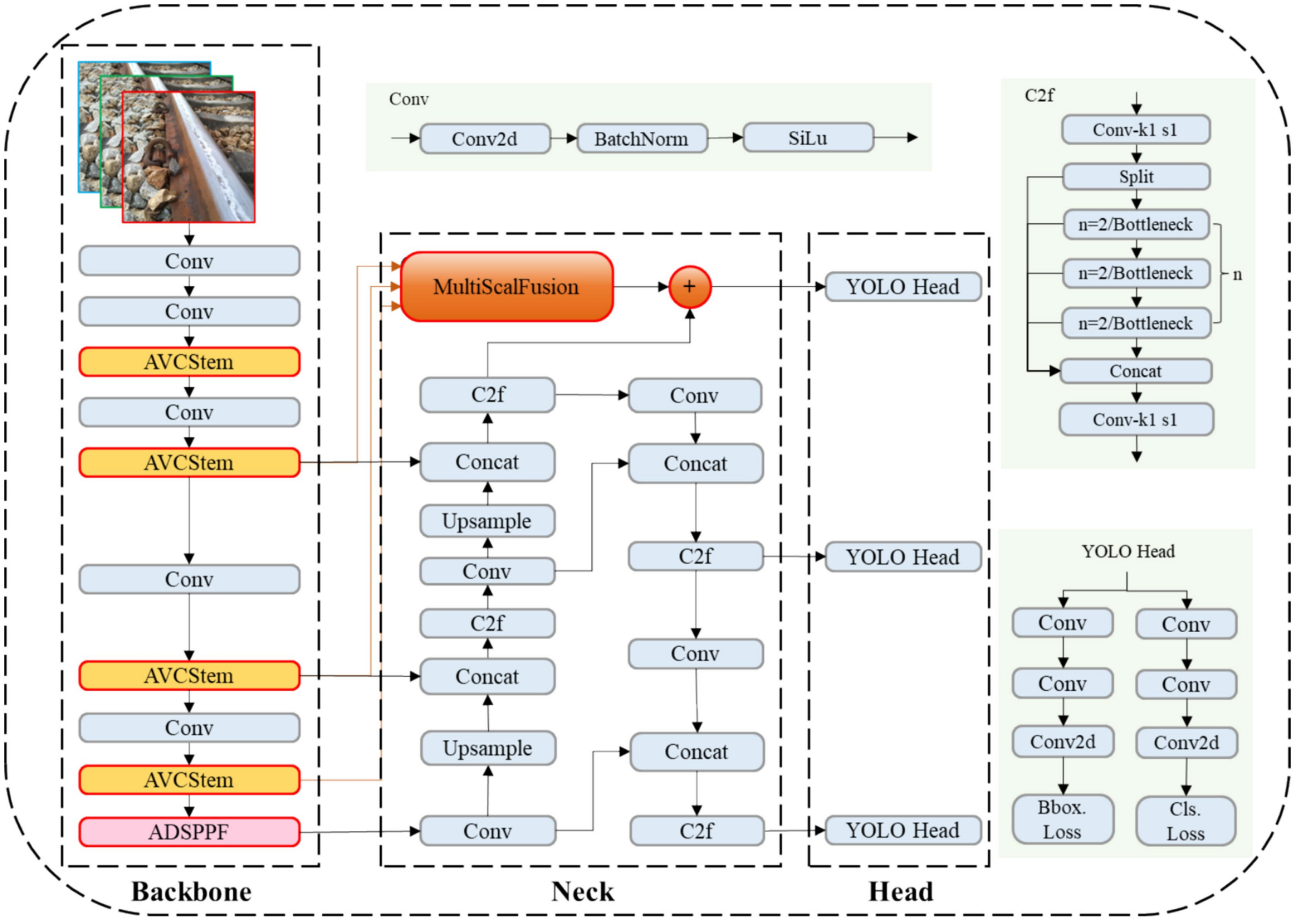
Improved YOLOv8 framework.

To address challenges related to fine-grained feature loss and multi-scale target recognition in rail defect detection, this study proposes an improved YOLOv8n network architecture. Figure 6 presents the architecture specifically designed to resolve these problems. Due to limitations in the original YOLOv8n backbone in capturing microscopic defects and adapting to targets of varying sizes, this study introduces the AVCStem module to replace the standard C2f module. By integrating the AKConv deformable kernel into the VoV-GSCSP structure, the module dynamically adjusts the receptive field, thereby significantly enhancing the network’s ability to represent rail stress cracks and missing bolts. Specifically, the AVCStem modules substitute the original C2f components in the backbone, enabling effective adaptation to defects with diverse morphologies under noisy or cluttered background conditions. Furthermore, to mitigate feature degradation during downsampling, an ADSPPF module based on the SENetV2 architecture is developed to replace the standard SPPF module. This module employs a multi-scale progressive pooling strategy, thereby achieving comprehensive feature preservation across a spectrum ranging from microscopic cracks to macroscopic fastener losses. Simultaneously, to enhance the transmission of minute defect features, a novel MSF fusion neck is designed. By combining the advantages of weighted feature fusion and a bidirectional pyramid architecture, it significantly improves the capability to capture fine-grained defect details. The proposed solution yields significant improvements in detection accuracy while preserving model compactness and computational efficiency, demonstrating its strong suitability for rail defect detection tasks in complex environments.

## 4 Experimental results and comparative analysis

### 4.1 Experimental environment

Experimental tests were carried out on two types of hardware to simulate the scenario of practical deployment: a server, which was used for model training, and edge devices, which served for real-time performance evaluation. Table 2 presents a summary of the specific configurations of the aforementioned hardware.

**Table 2:** Experimental environment configuration.

Hardware/software	Server workstation	Edge hardware (NVIDIA Jetson Nano)	Edge hardware (raspberry Pi 5)
Operating system	Ubuntu 22.04	Ubuntu 20.04 LTS (JetPack 5.1.2)	Raspberry Pi OS (Bookworm)
CPU	Intel(R) Core(TM) i9-14900KF @3.20 GHz	Quad-core ARM Cortex-A57 @1.43 GHz	Hexa-core ARM Cortex-A76 @2.4 GHz
GPU	NVIDIA GeForce RTX 4090 (24 GB VRAM)	NVIDIA Maxwell GPU (4 GB VRAM)	VideoCore VII NPU (1 TFLOPs)
RAM	32 GB DDR5	4 GB LPDDR4	8 GB LPDDR4
Software Stack	PyTorch 1.6, CUDA 12.1, Python 3.10	PyTorch 2.0, TensorRT 8.5	PyTorch 2.1, ONNX Runtime

### 4.2 Network training

**Figure 7:**
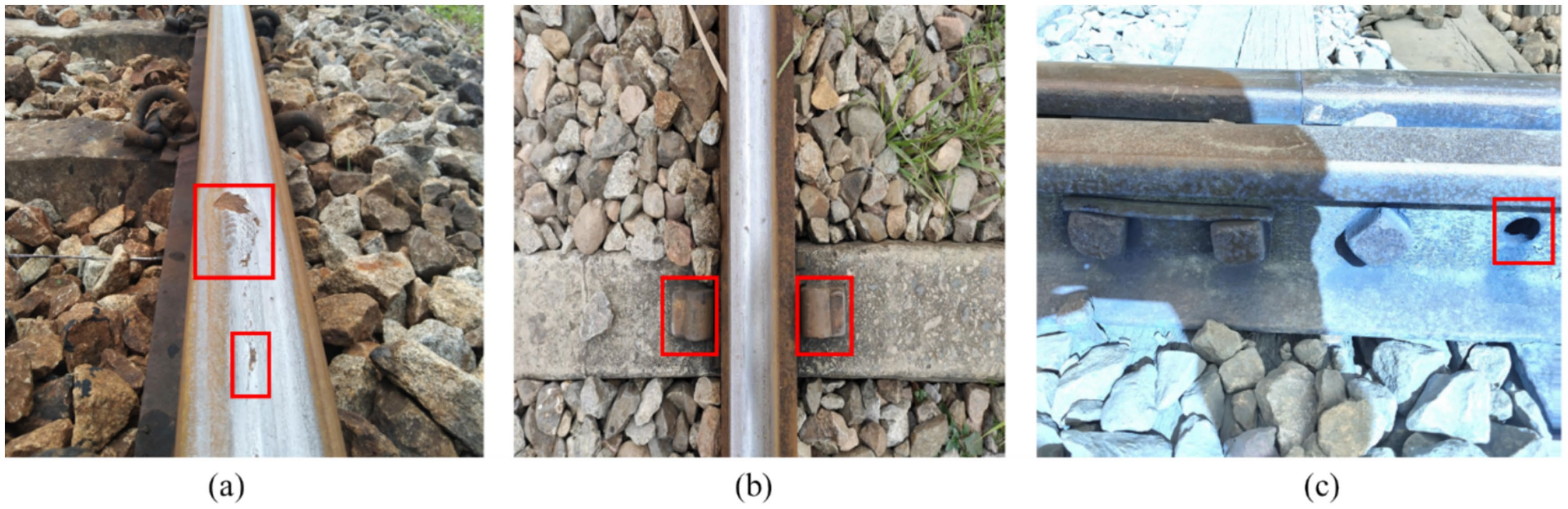
Track defect dataset: **(a)** surface damage; **(b)** missing buckles; **(c)** missing bolts.

In this study, a dataset comprising 2,129 images was constructed using the open-source Roboflow Universe dataset, augmented with real-world images collected by users. Among them, 1,500 images were used for training, 500 for validation, and 129 for testing. The dataset was reviewed and re-labeled by domain experts. As shown in Figure 7, the dataset includes defect types such as surface damage, missing bolts, and missing buckles. Before training, the model’s hyperparameters were configured. The training input image size was set to 640 × 640, with an initial learning rate of 0.01, updated using stochastic gradient descent (SGD). The momentum was set to 0.937, and the weight decay was 0.0005. During training, Mosaic data augmentation was employed to read multiple images simultaneously, which were then combined through inversion, scaling, and other transformations to enrich the detection background. Label smoothing was applied to prevent overfitting and improve the model’s generalization ability. Training was conducted over 300 epochs, with a batch size of 32 and 16 worker threads.

### 4.3 Performance indicators

In the evaluation framework of YOLO, the evaluation metrics for YOLO models are not standalone; rather, they serve as an integrative link connecting the “performance-efficiency-deployment” triad. Specifically, these metrics avoid the issue of “failure to achieve real-time inference” caused by focusing solely on detection accuracy while neglecting inference speed, and also prevent the problem of “hardware incompatibility” arising from prioritizing inference speed without considering model complexity. Consequently, when evaluating improved YOLO algorithms, the evaluation criteria are generally categorized into three dimensions: detection accuracy, model complexity, and real-time performance.

#### 4.3.1 Detection accuracy

To evaluate detection accuracy, commonly used indicators include precision (P), recall (R), and mean average precision (mAP). Specifically, P reflects the rate of false positives, R indicates the extent of missed detections, AP represents the area under the precision-recall (P-R) curve, and mAP is the mean of AP values, serving as a comprehensive measure of detector accuracy. The calculation formula is as follows (Formulas 9–12):


$$P=\frac{\mathrm{TP}}{\mathrm{TP}+\mathrm{FP}}$$ (9)

$$R=\frac{\mathrm{TP}}{\mathrm{TP}+\mathrm{FN}}$$ (10)

$$\mathrm{AP}=\int_{0}^{1}P(R)\mathrm{dR}$$ (11)

$$\mathrm{mAP}=\frac{1}{C}\sum_{i=1}^{C}AP_{i}$$ (12)

Here, TP denotes the number of true positive samples, FP denotes the number of false positive samples, and FN denotes the number of false negative samples.

#### 4.3.2 Model complexity

Model Complexity is the core metric connecting “model performance” and “practical deployment feasibility,” where its core directly determines the algorithm’s practicality in different hardware environments through Weight file size (MB), Trainable parameters (M), Floating-point operations per second (G) at 640 × 640 input (GFLOPs), and Peak GPU/CPU memory during inference (MB).

#### 4.3.3 Real-time performance

Real-time performance serves as a core metric for evaluating the inference efficiency and temporal responsiveness of a model. Its essence lies in quantifying the processing speed and operational stability of the model when executing detection inference on input images or video frames under a specific hardware configuration. In this study, inference experiments were carried out on two typical edge computing platforms, namely NVIDIA Jetson Nano and Raspberry Pi 5, with a consistent batch size of 1 configured to validate the frames per second (FPS) of the improved YOLO model.

### 4.4 Training curve

**Figure 8:**
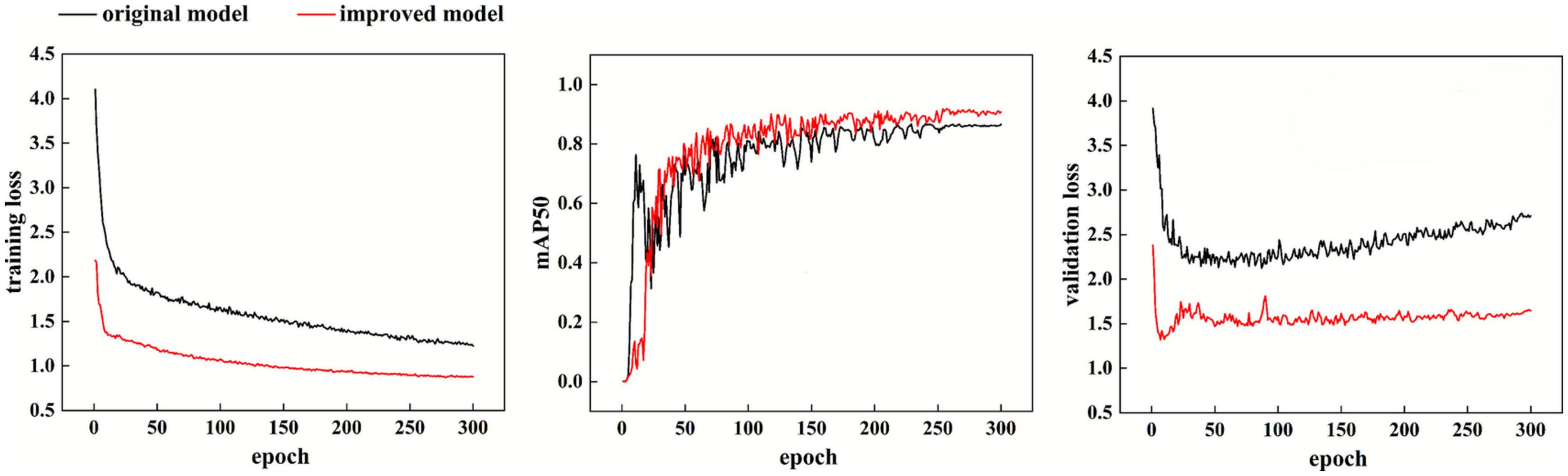
Loss function curve.

To intuitively visualize the enhancements achieved by the improved algorithm, the training curves are presented. Figure 8 illustrates the training loss, mAP50, and validation loss curves for both the original and improved models after 300 training epochs. Notably, the training process for the original model terminates after 200 epochs due to a lack of further improvement in accuracy. In contrast, the improved model demonstrates accelerated convergence, yielding predictions that are closer to the ground truth. Moreover, the mAP50 metric shows significant improvement, as clearly illustrated by the curves. These observed enhancements provide strong evidence of the effectiveness of the proposed algorithm. The validation loss curve reveals the model’s generalization capability. The original model’s validation loss gradually increases in the later training stage, which implies overfitting. In contrast, the improved model’s validation loss remains low and stable throughout training, demonstrating that the proposed improvements effectively mitigate overfitting and enhance the model’s robustness on unseen data.

### 4.5 Ablation experiment

To assess the performance improvements achieved by integrating three optimization strategies—the AVCStem module, ADSPPF module, and MSF module—into YOLOv8n, a series of ablation experiments was conducted on the dataset. The evaluation metrics included model size, parameter count, precision (P), recall (R), mean average precision at an IoU threshold of 0.5 (mAP@0.5), and mean average precision across the IoU range of 0.5–0.95 (mAP@0.5:0.95) (Ni et al., 2021). These experiments evaluated various combinations of the aforementioned modules to isolate their individual and collective contributions. The corresponding experimental results are summarized in Table 3.

**Table 3:** Ablation experiment.

Methods				Model size/MB	Params/M	P/%	R/%	mAP@0.5/%	mAP@0.5:0.95/%
YOLOv8n	AVCStem	ADSPPF	MSF						
√				6	2.68	87.9	79.0	85.3	64.5
√	√			5.1	2.4	88.7	78.0	86.2	61.6
√		√		5.7	2.72	87.4	82.0	86.3	65.6
√			√	5.7	2.7	88.9	78.2	86.2	64.6
√	√		√	5.2	2.41	87.6	86.7	88.1	66.2
√	√	√	√	5.2	2.45	90.2	84.5	**90.2**	**73.2**

Bold values indicate the optimal performance of the corresponding metrics in the ablation experiments.

According to Table 2, integrating the AVCStem module into the YOLOv8n framework results in a 0.8 and 0.9% increase in P and mAP@0.5, respectively, while the model size and parameter count decrease by 15 and 10.45%. However, R and mAP@0.5:0.95 show a slight decline, suggesting that while AVCStem improves localization accuracy, it may inadvertently filter out some positive samples. With the addition of the ADSPPF module, R increases significantly to 82.0%, accompanied by slight improvements in both mAP@0.5 and mAP@0.5:0.95, indicating broader object coverage and enhanced bounding box regression accuracy. However, a slight decrease in precision may result from an increase in false positives introduced by the more aggressive detection strategy. The MSF module significantly improves accuracy to 88.9%, while maintaining comparable mAP@0.5 and mAP@0.5:0.95 values relative to the baseline model. This suggests that the MSF module enhances feature representation, thereby reducing misclassification. The combination of AVCStem and MSF yields notable improvements in R (86.7%) and mAP@0.5 (88.1%). These improvements demonstrate that AVCStem enhances low-level feature extraction, whereas MSF improves multi-scale feature integration, contributing to more robust detection performance. Full integration of all three modules results in the best overall performance, achieving 90.2% precision, 90.2% mAP@0.5, and 73.2% mAP@0.5:0.95, along with a reduced model size (5.2 MB) and a comparable parameter count. These results underscore the strong complementarity among the proposed modules.

**Figure 9:**
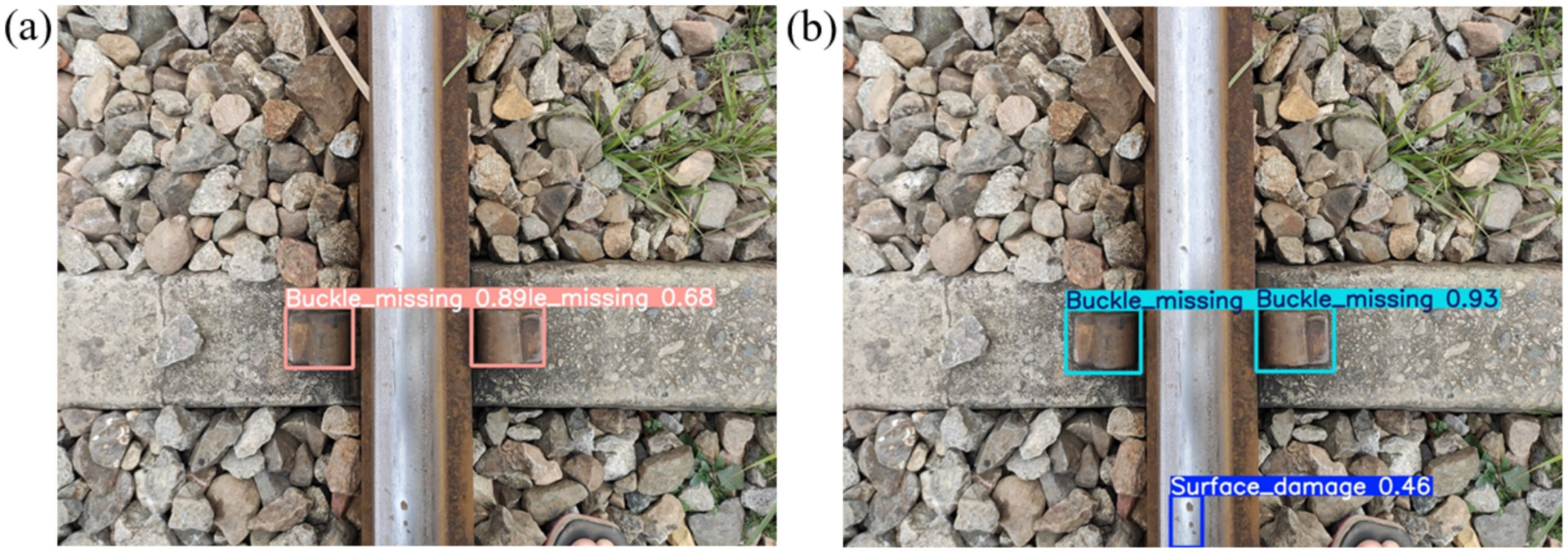
Comparison of detection effects: **(a)** the original YOLOv8n algorithm; **(b)** improved YOLOv8n algorithm.

As shown in Figure 8, the detection performance comparison demonstrates significant improvements. Figure 9a illustrates the original YOLOv8n algorithm, which exhibits limitations in detecting small or blurred targets, potentially resulting in missed or false detections. In contrast, Figure 9b showcases the enhanced YOLOv8n algorithm proposed in this study. This improved approach effectively addresses the aforementioned challenges, successfully identifying missing fasteners while simultaneously detecting minute defects on the rail surface with precision. Ablation studies confirm that each proposed module contributes to performance improvement when used independently, while their combination leads to substantial gains in both detection accuracy and efficiency. The improved YOLOv8n model not only surpasses the baseline across all major evaluation metrics but also maintains a lightweight architecture, making it well-suited for deployment in resource-constrained railway detection environments.

**Figure 10:**
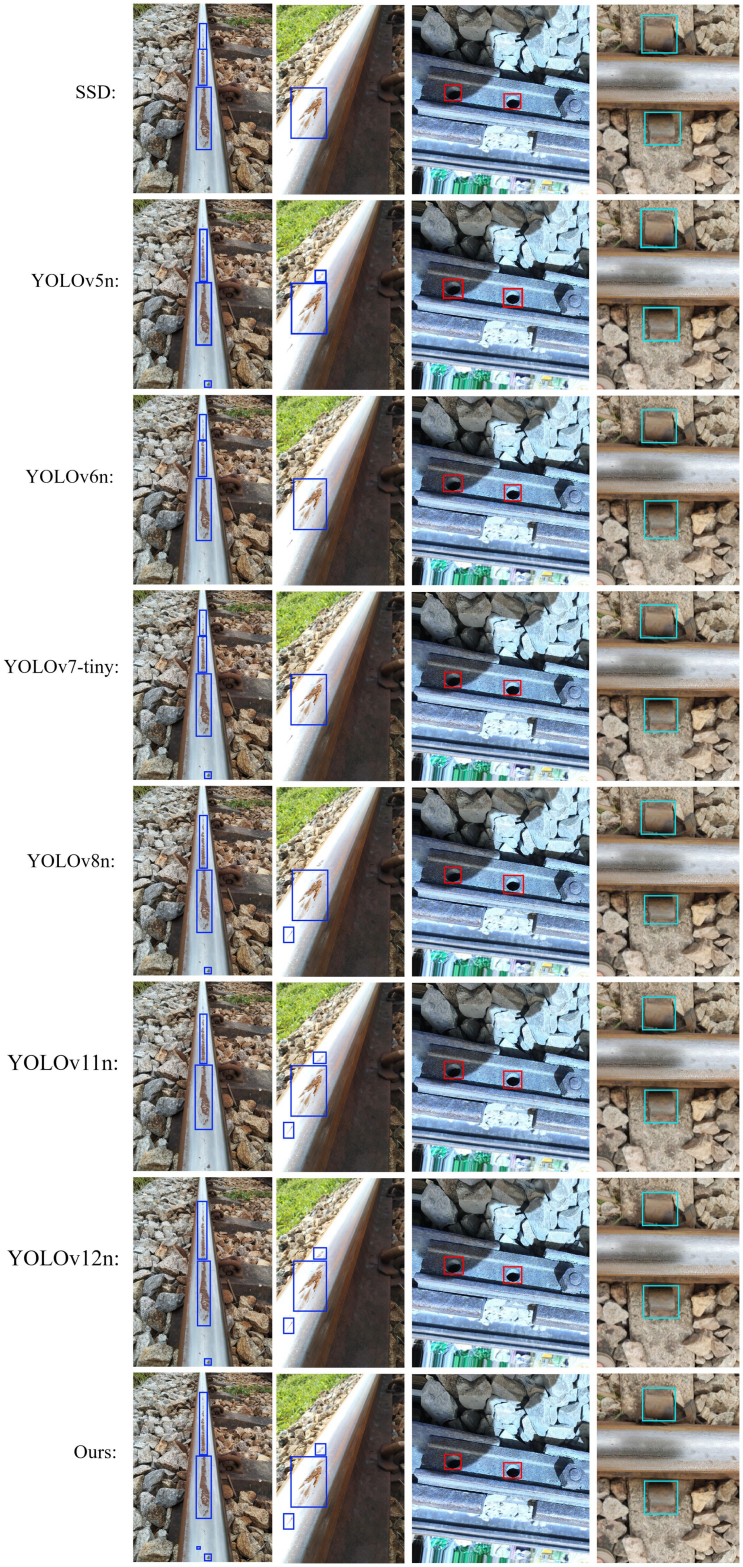
Comparison chart of the effect of detection.

### 4.6 Module-level complexity

To analyze the incremental complexity of each module, this study measure the parameters, GFLOPs, and inference latency of the original and improved modules (Jetson Nano, batch = 1) (Table 4).

**Table 4:** Module-level complexity and timing profile.

Module type	Original module	Improved module	ΔParams (M)	ΔGFLOPs (G)	∆Latency (ms)
Backbone (per module)	C2f (0.82 M)	AVCStem (0.73 M)	−0.09	−0.12	−1.4
Backbone (total 3 modules)	2.46 M	2.19 M	−0.27	−0.36	−4.2
Neck (SPPF)	SPPF (0.068 M)	ADSPPF (0.074 M)	+0.006	+0.15	+2.1
Neck (FPN, per module)	FPN Conv (0.15 M)	MSF PConv (0.11 M)	−0.04	−0.07	−1.2
Neck (total 3 FPN modules)	0.45 M	0.33 M	−0.12	−0.21	−3.6
Overall model	–	–	−0.23	−0.42	−5.7

As shown in Table 4, each AVCStem reduces parameters by 0.09 M and latency by 1.4 ms, with total backbone savings of 0.27 M params and 4.2 ms latency—driven by GSConv and residual compression. ADSPPF increases parameters by 0.006 M and latency by 2.1 ms but provides critical gains in illumination robustness and fine-feature retention (justified by ablation results). Each MSF module reduces parameters by 0.04 M and latency by 1.2 ms, with total neck savings of 0.12 M params and 3.6 ms latency—due to PConv’s reduction of redundant computations. The overall model reduces parameters by 0.23 M (8.6%), GFLOPs by 0.42G (7.7%), and latency by 5.7 ms (13.5%)—confirming that the improved modules enhance accuracy without sacrificing efficiency.

### 4.7 Quantitative evaluation of real-time performance

To substantiate the “real-time” claim, this study measure end-to-end latency, FPS, GFLOPs, and memory usage on both server and edge hardware (Table 5).

**Table 5:** Real-time performance metrics (640 × 640, batch = 1).

Hardware	Model	Latency (ms)	FPS	GFLOPs (G)	Memory Usage (MB)	Size (MB)	Params (M)
NVIDIA RTX 4090	YOLOv8n	9.5	105.3	5.2	486	5.6	2.68
	Ours	8.2	121.9	4.8	423	5.2	2.45
NVIDIA Jetson Nano	YOLOv8n	42.1	23.7	5.2	392	5.6	2.68
	Ours	35.0	28.6	4.8	345	5.2	2.45
Raspberry Pi 5 (NPU)	YOLOv8n	58.3	17.1	5.2	289	5.6	2.68
	Ours	49.2	20.3	4.8	256	5.2	2.45

As shown in Table 5, on Jetson Nano (a typical edge AI device), the improved model achieves 28.6 FPS—exceeding the real-time threshold (≥20 FPS) for railway inspection robots. Latency is reduced by 16.9% compared to the baseline, thanks to the lightweight design of AVCStem and MSF. The improved model’s GFLOPs (4.8G) are 7.7% lower than the baseline (5.2G), and memory usage is reduced by 12.0–14.5% across all hardware—critical for resource-constrained edge devices.

### 4.8 Comparative experiment

To verify the performance advantages of the improved YOLOv8n (denoted as Our) in the task of orbital defect detection, as shown in Figure 10, seven types of mainstream lightweight object detection algorithms, namely SSD, YOLOv5n, YOLOv6n, YOLOv7-tiny, YOLOv8n, YOLOv11n, and YOLOv12n, were selected for comparison. Comparative experiments were conducted from six dimensions: Precision (P), Recall (R), mAP50, mAP50-95, model weight, and number of parameters, with the results presented in Table 6.

**Table 6:** Comparative experimental results.

Model	P(%)	R(%)	mAP50(%)	mAP50–95(%)	Weights (MB)	Parameters (M)
SSD	80.2	70.3	82.1	–	15.3	3.81
YOLOv5n	89.9	79.9	84.8	62.5	3.7	1.76
YOLOv6n	84.1	74.9	82.6	53.9	8.6	4.16
YOLOv7-tiny	85.0	85.4	85.9	63.3	12.3	6.01
YOLOv8n	87.9	79.0	85.3	64.5	5.6	2.68
YOLOv11n	86.7	86.3	88.1	69.5	5.5	2.58
YOLOv12n	91	74.2	84.7	60.9	5.5	2.51
Our	90.2	84.5	**90.2**	**73.2**	5.2	2.45

Bold values indicate the optimal performance of the corresponding metrics among all compared models.

From the perspective of detection accuracy, the improved model exhibits significant advantages in core metrics: the mAP50 of the Our model reaches 90.2%, which is 2.1 percentage points higher than that of the second-best YOLOv11n (88.1%) and 4.9 percentage points higher than the original YOLOv8n (85.3%), making it the only algorithm among all comparative models that exceeds 90%. In terms of the mAP50-95 metric, the Our model leads substantially with a score of 73.2%, which is 3.7 percentage points higher than YOLOv11n (69.5%) and 8.7 percentage points higher than the original YOLOv8n (64.5%), indicating that the improved model achieves optimal robustness in detecting orbital defects under different IoU thresholds. Regarding precision, the Our model achieves 90.2%, second only to YOLOv12n (91.0%), while its recall rate reaches 84.5%, far exceeding YOLOv12n’s 74.2%. This effectively balances precision and recall, reducing the risks of missed detection and false detection of orbital defects.

From the perspective of lightweight deployment, the Our model balances performance and deployment efficiency: with only 2.45 million parameters, it has the smallest parameter count among all comparative models, representing a 2.39% reduction compared to YOLOv12n (2.51 M), an 8.58% decrease compared to the original YOLOv8n (2.68 M), and a 59.23% reduction compared to YOLOv7-tiny (6.01 M). The model weight is 5.2 MB, slightly lower than YOLOv11n/YOLOv12n (5.5 MB) and much lower than models such as SSD (15.3 MB) and YOLOv7-tiny (12.3 MB), meeting the lightweight deployment requirements of edge devices for orbital inspection.

Overall, as a traditional object detection algorithm, SSD is at a disadvantage in both performance and lightweight aspects and is no longer suitable for the orbital defect detection scenario. Among the lightweight models of the YOLO series, YOLOv11n achieves a preliminary balance between performance and lightweight design but is still inferior to the Our model. While maintaining lightweight advantages, the improved YOLOv8n breaks through the bottleneck of “high performance accompanied by high parameter count” in existing algorithms, achieving dual optimality of detection accuracy and deployment efficiency in the orbital defect detection task.

The proposed improved YOLOv8n model significantly outperforms mainstream lightweight detection models with respect to accuracy, robustness, and computational efficiency. Its superior mAP metrics and compact architecture demonstrate strong potential for real-time deployment in railway track defect detection systems, where both accuracy and speed are critical.

## 5 Conclusion

This paper proposes a lightweight improved YOLOv8n detection model that integrates AVCStem, ADSPPF, and MSF modules, significantly enhancing the accuracy and efficiency of railway track defect detection. By incorporating variable convolution, a cross-scale feature retention mechanism, and a multi-branch fusion strategy, the model outperforms existing mainstream detection frameworks across multiple key metrics. The experimentally validated model not only maintains lightweight characteristics—including compact size and low parameter count—but also demonstrates the capability to accurately detect tiny defects in complex environments. In the future, efforts will focus on deploying this model on track inspection robots and edge AI devices, as well as integrating it with video sequence modeling to enable dynamic defect analysis of continuous tracks.

## Data Availability

The original contributions presented in the study are included in the article/supplementary material, further inquiries can be directed to the corresponding author.
